# Control strategy of hand movement depends on target redundancy

**DOI:** 10.1038/srep45722

**Published:** 2017-03-31

**Authors:** Shunta Togo, Toshinori Yoshioka, Hiroshi Imamizu

**Affiliations:** 1 Graduate School of Informatics and Engineering, The University of Electro-Communications, Tokyo, Japan; 2 Cognitive Mechanisms Laboratories, Advanced Telecommunications Research Institute International, Kyoto, Japan; 3 Department of Psychology, The University of Tokyo, Japan

## Abstract

Reaching toward a point target has been intensively studied in human motor control. However, little is known about reaching toward a redundant target, such as grasping a bar, in which the grasping point is irrelevant to the achievement of a task. We examined whether humans could solve the target-redundancy and control problems in a serial fashion or control their body without solving the target-redundancy problem. We equalized the target ranges between two reaching tasks: a point-to-point reaching task without target-redundancy and a point-to-bar reaching task with target-redundancy. In the both tasks, we measured hand viscoelasticity at movement end as parameters that reflect the adopted control strategy. As a result, the hand viscoelasticity in the point-to-bar reaching task was smaller than that in the point-to-point reaching task, even under the same kinematics. These results indicate that the hand viscoelasticity was modulated depending on the target-redundancy. Moreover, it is suggested that a human reaches toward a redundant target by effectively utilizing information of target redundancy rather than explicitly solving the target-redundancy problem.

When we move our hand to some specific target, we plan how to reach it before performing a hand movement^1^. In the traditional point-to-point reaching paradigm^2^,^3^, subjects are explicitly instructed where they should reach in a workspace. For example, when we pick up a ball, we have to move our hand toward some specific point in three-dimensional space. Therefore, the central nervous system (CNS) needs to plan how to move the hand toward the given specific target point. By contrast, when the target is given as a “bar” instead of a “point”, subjects can move their hand to anywhere on the bar to achieve the task. In this case, movements along the bar need not be controlled. For example, when we grab a vertical pole, we have to control the hand movements in the plane orthogonal to the pole, but we need not control the movements along the pole. Such a point-to-bar reaching paradigm (manifold reaching paradigm^4^,^5^) contains a problem in which the CNS has to deal with the target redundancy, i.e., where should we move our hand? By comparing point-to-point reaching with point-to-bar reaching, we can gain an insight into how the CNS deals with a target redundancy in movement planning.

We hypothesized two types of strategies adopted by the CNS to deal with the target redundancy in the point-to-bar reaching task. One is a strategy that explicitly solves the target redundancy problem. In this strategy, the CNS explicitly determines a specific target point on the bar to transform the point-to-bar reaching problem to the point-to-point one. After solving the redundancy problem, the CNS can use the same control strategy in both point-to-bar and point-to-point reaching movements. An alternative type is a strategy that does not explicitly solve the target redundancy problem. In this case, the CNS temporarily holds information of a target redundancy and utilizes it for the planning and execution of a point-to-bar reaching movement. Therefore, the CNS adopts different strategies to perform the point-to-bar and point-to-point reaching tasks. This implies that the CNS utilizes the information of target redundancy to generate a motor command. In this study, we investigated which type of strategy the CNS adopts to perform the point-to-bar reaching task. In other words, this study investigated whether the CNS explicitly solves the target redundancy problem during movement planning.

We have to control movement conditions to compare the difference in control strategies from the kinematics. In this study, we controlled experimental environment, start position of movement, target position, required movement precision, and movement duration between two reaching tasks and equalize kinematics between the tasks. Previous studies have indicated that humans voluntarily control their viscoelasticities (i.e., movement dynamics) according to the goal of the given task^6^,^7^,^8^,^9^. In particular, Lametti *et al*. reported that the limb stiffness was modulated when the shape of the target changed^9^. Their study indicates that the geometric property of target affects hand viscoelasticity. Because target redundancy can be considered one of the geometric properties of the target, we hypothesized that if the subject changes their control strategy according to the task goal (with or without target redundancy), then the hand viscoelasticity is different between the two tasks, even under the same kinematics. In movement planning for the two tasks, the stability at movement end is important, because subjects were required to conduct ballistic movements in which the required precision of the movement was controlled. Therefore, we assumed that the difference in hand viscoelasticity between the two tasks would be greater around movement end than during the movement. In this study, we measured and compared the hand viscoelasticities at movement end between the point-to-bar and point-to-point reaching tasks. Evaluating the difference in hand viscoelasticity between the two tasks, we revealed which type of strategy the CNS adopted to perform the point-to-bar reaching task, i.e., whether the CNS solved the target redundancy problem in movement planning.

## Results

**Figure 1:**
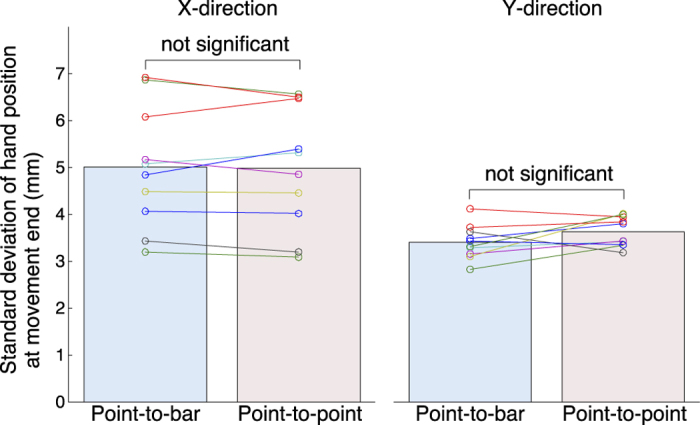
Standard deviations of hand position at movement end of all subjects. Standard deviations between task conditions are not significantly different (evaluated by paired *t*-test).

To properly compare control strategies from motor outputs, we controlled the movement kinematics. First we check the movement kinematics in both reaching tasks. The subjects showed similar amplitude of variability of hand position at movement end in both reaching tasks (Fig. 1, a paired *t*-test showed no significant difference in the *X*-direction: *t*_(9)_ = 0.26, *P* = 0.80 or the *Y*-direction: *t*_(9)_ = −1.74, *P* = 0.12). These results indicate that we could properly control the movement kinematics in both reaching tasks.

**Figure 2:**
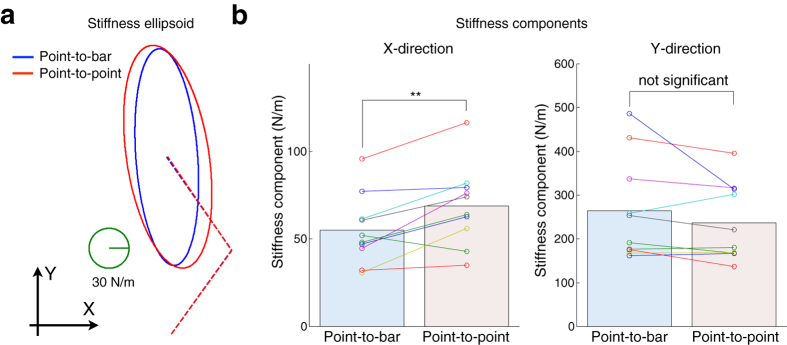
Hand stiffness at movement end. (**a**) Stiffness ellipsoids of a typical subject. Solid-line ellipsoids indicate hand stiffness, while dashed lines denote arm postures. (**b**) Stiffness components along *X*- and *Y*-directions of all subjects (*n* = 10). Differences in stiffness component between task conditions are statistically evaluated by paired *t*-test (**P < 0.01).

**Figure 3:**
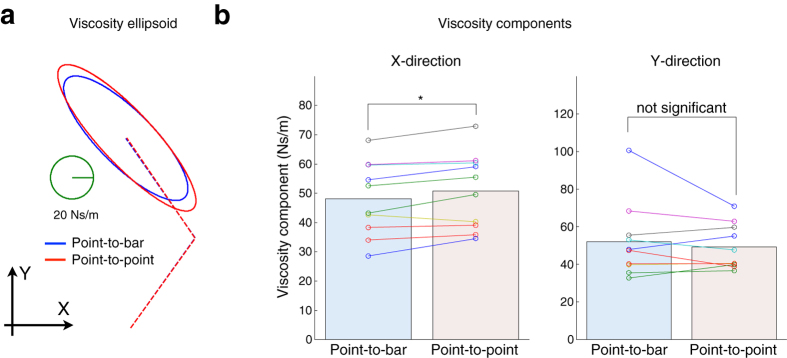
Hand viscosity at movement end. (**a**) Viscosity ellipsoids of a typical subject. Solid-line ellipsoids indicate hand viscosity, while dashed lines denote arm postures. (**b**) Viscosity components along *X*- and *Y*-directions of all subjects (*n* = 10). Differences in viscosity component between task conditions are statistically evaluated by paired *t*-test (*P < 0.05).

Next we check the movement dynamics. Figure 2 shows that the hand stiffness was different between the two reaching tasks. The stiffness ellipsoid of a typical subject in the point-to-bar task was smaller than that in the point-to-point task (Fig. 2a). According to all subjects (*n *=* *10), the stiffness component along the *X*-axis in the point-to-bar task was significantly smaller than that in the point-to-point task (Fig. 2b, paired *t*-test: *t*_(9)_ = −3.59, *P* = 0.0058 < 0.01). However, the stiffness component along the *Y*-axis was not significantly different between the two tasks (*t*_(9)_ = 1.50, *P* = 0.17). Moreover, the hand viscosity showed similar results for hand stiffness (Fig. 3). Specifically, the viscosity ellipsoid of a typical subject in the point-to-bar task was smaller than that in the point-to-point task (Fig. 3a). According to all subjects (*n *= 10), the viscosity component along the *X*-axis in the point-to-bar task was significantly smaller than that in the point-to-point task (Fig. 3b, a paired *t*-test: *t*_(9)_ = −3.10, *P* = 0.013 < 0.05). However, the viscosity component along the *Y*-axis was not significantly different between the two tasks (*t*_(9)_ = 0.84, *P* = 0.42 > 0.05). These results indicate that subjects modulated hand viscoelastic properties along only the *X*-axis, which is switched from the redundant direction to the control direction depending on the task.

## Discussion

In this study, we examined how the CNS deals with the target redundancy, i.e., the target redundancy problem is explicitly solved or not. In the point-to-point reaching, the CNS deals with the hand trajectory redundancy^10^, the effector redundancy^11^, the joint redundancy^12^, and the muscle redundancy^13^. However, we could not approach the target redundancy problem only by the traditional point-to-point reaching paradigm. Therefore, we compared the dynamical motor properties, the hand viscoelasticities, between the point-to-bar and point-to-point reaching tasks. Our results show that hand viscoelasticities were different between two reaching tasks (Figs 2 and 3), reflecting how subjects adopted different control strategies depending on the target redundancy. This supports the hypothesized strategy in which the CNS does not explicitly solve the target redundancy. These results imply that the CNS plans and executes reaching movement by utilizing information of task redundancy rather than sequentially solving the target redundancy problem.

Previously, it has been reported that the voluntary modulation of hand viscoelasticity was affected by the dynamics of the environment^6^,^7^ and required task accuracy^8^,^9^. Since, in our study, dynamics of experimental environment was constant across the tasks, these effects unlikely affected the results. Lametti *et al*. reported that the shape of the target, i.e., the required movement precision, affects the limb stiffness^9^. In our current study, we controlled the required movement precision. Therefore, both studies found that the geometric property of the target affects hand viscoelasticity. However, the geometric property in this study was target redundancy, while that in the earlier study was required movement precision. In our study, the size of the target on the screen was different between the two tasks. If the size of the target on the screen had affected the control of movements in the *X*-direction, the variability of hand position at movement end in the point-to-point task would be smaller than that in the point-to-bar task due to the small size of the target. However, there was no significant difference in the standard deviation of hand position at movement end between the two tasks (Fig. 1). We set the target length along the *X*-direction to ±2 standard deviation of hand position at movement end in the point-to-bar task, and this length was probably sufficient for a natural reach to the target. Therefore, the difference in the target size between the tasks did not affect the hand positions at movement end and was unlikely the direct cause of the change in stiffness and viscosity components along the *X*-direction (Figs 2 and 3). In addition, decreases in joint stiffness due to fatigue^14^ and motor learning^15^ have been reported. In this study, subjects arbitrarily took short break to avoid their fatigue, and enough practiced both reaching tasks before experiment begun. Moreover, the point-to-bar reaching task in which the hand viscoelasticity was smaller was performed before the point-to-point reaching task. Therefore, the effects of fatigue and the motor learning also unlikely affected the results. Because these previously reported effects unlikely explain our results, we came to conclusion that the hand viscoelasticity is modulated depending on the target redundancy. The hand viscoelasticity in the redundant direction was smaller than that in the control direction. This suggests that the CNS discriminates the redundant direction in the workspace and directionally modulates hand viscoelasticity.

The viscoelastic component in the redundant direction may be reduced to save movement energy and to minimally intervene in correction of movement error^16^,^17^. Since the given perturbation had quite small amplitude, brief duration and no visual information, it would be difficult to accurately perceive the direction of the perturbation. According to a previous study, a time window of 20–45 ms after perturbation onset corresponds to a short latency stretch response, and 105 ms after corresponds to an early voluntary response^18^. We calculated the hand viscosity at the first peak of hand force (28.1 ± 0.77 ms after perturbation onset) and found that the calculated hand viscosity component was significantly different. Therefore, the directional modulation of hand viscoelasticity was unlikely to reflect somatosensory feedback response to external perturbation but rather to reflect pre-adjustment of feedback gain. Since subjects repeatedly performed the two reaching tasks, they could judge whether they should correct their movements depending on target redundancy. We controlled the mean hand position at movement end in both tasks and fixed the subjects’ wrist and body, which indicates that the geometric property of subject’s arm was controlled. Therefore, we thought that the difference in viscoelastic property reflects the pre-adjustment of feedback gain, and that the adjustment of feedback gain would be caused by adjustment of the sensitivity of muscle spindle and/or the co-contraction level of the agonist and antagonist muscles. Nahsed *et al*. indicated that the feedback response to an external perturbation during reaching movement was affected by the shape of the target, i.e., target reduncdancy^5^. In the Discussion section of their paper, they predicted that the feedback responses based on target shape were also present during unperturbed reaching movements. In our study, both reaching tasks were sufficiently practiced, and we did not apply an external perturbation during movements. However, we controlled movement kinematics, so the hand viscoelasticities were significantly different. These results suggest that the CNS modulates feedback gain based on the target redundancy, even without needing a response to the external perturbation. Therefore, our results support the prediction by Nashed *et al*. Our study and the earlier study together suggest that the CNS modulates a gain of feedback response based on the target redundancy independent of the external perturbation.

Tagliabue, *et al*. reported that (1) the response to the external perturbation was not modulated based on target redundancy, and (2) no directional modulation of hand stiffness was observed^19^. With respect to the first report, our^20^ study as well as the earlier studies^5^,^11^,^21^ reported that the response to external perturbation was modulated depending on the redundant direction. Therefore, the majority of the previous studies have supported the hypothesis that the CNS modulates the response to the perturbation depending on the target redundancy. With respect to the second reported conclusion, earlier studies suggested that the CNS directionally modulates hand stiffness depending on the shape of the target and the direction of the force field^9^,^22^. Therefore, the modulation of hand viscoelasticity depending on the redundant direction observed in this study was a reasonable result.

## Methods

### Subjects

Ten healthy right-handed males participated in the experiments. Their average age was 25.8 (20–33 years). Their dominant hand was assessed by the Edinburgh handedness inventory^23^. The experiments were approved by the ethics committee at Advanced Telecommunication Research Institute International, and the experimental protocol was carried out in accordance with the latest version of the Declaration of Helsinki. All subjects received explanations about the experimental procedure and gave their written informed consent.

### Apparatus

**Figure 4:**
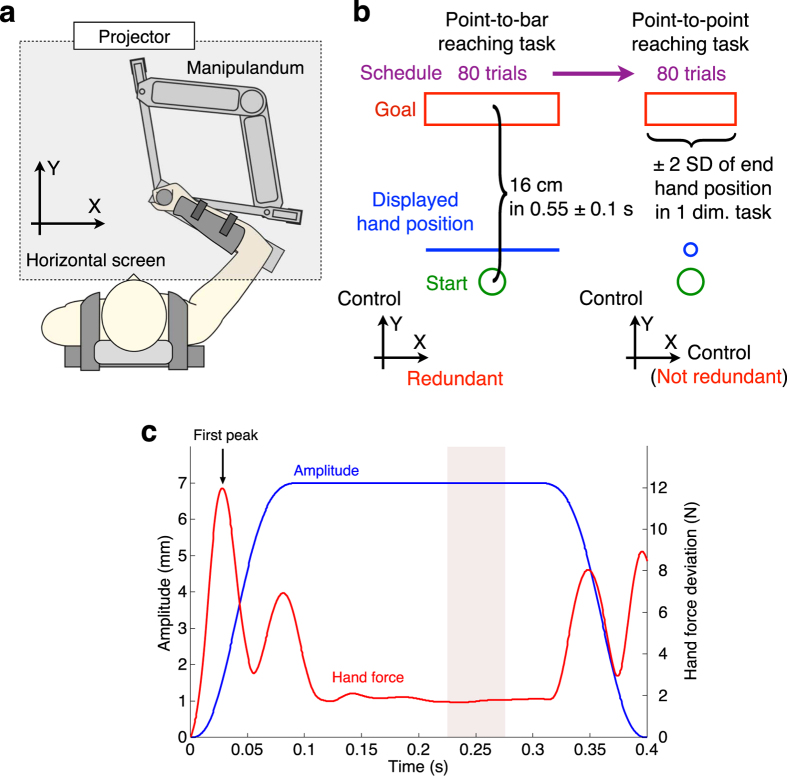
Methods and task conditions. (**a**) Subjects sit on a chair while grasping the handle of a manipulandum. The projector displays the target and the subject’s hand position. The manipulandum applies brief and small-amplitude perturbation to the subject’s hand at movement end. Hand force and position are recorded and used to estimate hand viscoelasticity. (**b**) Task conditions and displayed information for subjects. In both conditions, subjects move their hand along the *Y*-direction, which is thus the control direction. However, whether the *X*-direction is redundant or not depends on the target dimensionality. (**c**) Time profile of positional perturbation and mean deviation of tangential hand force of a typical subject. The amplitude of perturbation smoothly increases up to 0.1 s through a sixth-order polynomial (blue line). The 7-mm amplitude is maintained for 0.2 s, and then the amplitude smoothly decreases. Hand force increases in the ramp-up phase and then stabilizes (red line). First peak of hand force appears at 28.1 ± 0.77 ms after perturbation onset (across subjects). Mean deviation data in the pink shaded area are used to estimate a hand stiffness matrix (from 0.2255 s to 0.2755 s). The deviation data at first peak of hand force are used to estimate a hand viscosity matrix.

We used a manipulandum system named “twin visuomotor and haptic interface system (TVINS)” to record force and movement kinematics of both hands at 2000 Hz (Fig. 4a). Subjects sat on an adjustable chair while grasping the handle of TVINS. The subject’s forearm was secured to a support beam on the horizontal plane by a plastic cuff. The plastic cuff also restricted the subject’s arm movements to shoulder and elbow movements. TVINS consists of two parallel-linked, direct-drive floating manipulanda using air magnets. In this study, we used only one manipulandum. The position of the subject’s hand was projected on a horizontal screen placed above the mechanical plane and below shoulder level. The screen prevented the subjects from directly seeing their arm. A 6-axis force sensor attached to the handle of TVINS recorded hand force. The position of the manipulandum was measured using optical joint position sensors.

### Task procedures

Subjects performed the point-to-bar reaching task with the one-dimensional target for 80 trials first, then the point-to-point reaching task with the two-dimensional target for 80 trials (Fig. 4b). In the point-to-bar reaching task, only the position of the subject’s hand in the *Y*-direction was fed back. Thus, subjects needed to control only the *Y*-directional movement. The target bar and subject’s hand were displayed by a 1.5 cm × 19 cm rectangle and a 2 mm × 19 cm rectangle (line), respectively. In the point-to-point reaching task, the hand positions in both directions were fed back. Thus, subjects had to control both directional movements. To equalize the target ranges between the two reaching tasks, we determine a target length in the *X*-direction in the point-to-point task as the standard deviation of hand position at movement end in the point-to-bar task. This enabled us to control the kinematic properties of movements in the point-to-point task to those in the point-to-bar task. Specifically, the target point width along the *Y*-axis was 1.5 cm, as with the point-to-bar reaching task, but the target length along the *X*-axis was ± 2 standard deviation of hand position at movement end in the point-to-bar task (mean ± SD: 3.13 ± 0.75 cm). The position of the subject’s hand was displayed by a 4-mm-diameter circle. The start position was displayed by a 1-cm-diameter circle placed 37 cm ahead of the center of gyration of the shoulder. The center of the target in the point-to-bar task was 16 cm ahead of the start position. The center in the point-to-point task was mean hand position at movement end in the point-to-bar task. We defined the times at movement start and end as the points when the tangential hand velocity was above and below 0.02 m/s, respectively.

Under both task conditions, subjects were instructed to move their hand 16 cm toward the target in 0.55 ± 0.1 s. In the point-to-bar task, they were instructed to move the line within the box and not to give attention to hand movements in the *X*-direction. They were not explicitly instructed where they should move the hand on the bar. In the point-to-point task, they were instructed to move the point within the box and to apply control of movements in both directions.

To estimate the hand viscoelasticity, we applied a positional perturbation to the subjects’ hands by using the TVINS at movement end (the time when the tangential velocity of the hand was smaller than 0.02 m/s) and measured reactive force. The position of the manipulandum handle automatically shifted toward eight directions in a pseudo-random order. Each perturbation had small amplitude (7 mm) and short duration (0.4 s) with ramp-up, hold and ramp-down profiles (Fig. 4c). To minimize mechanical vibration, short transition (ramp-up and ramp-down) phases were achieved by using a sixth-order polynomial with zero velocity and zero acceleration at the boundaries and zero end jerk^24^. The perturbation was applied in every trial. In both tasks, subjects knew that the perturbation was applied to their hands only at movement end but could not predict the direction of perturbation. During perturbation, no visual feedback of the hand position was given to the subjects. Before the experiments began, subjects practiced both tasks to get used to the tasks and hand movements with TVINS.

## Data analysis

The recorded hand position and hand force data were all filtered with a fourth-order Butterworth low-pass filter with a 40-Hz cutoff frequency. Using the conventional method^24^, we estimated the hand viscoelasticity^25^,^26^ based on using the subjects’ reaction force. We obtained hand velocity and acceleration by calculating the numerical differential of the measured hand position. To remove outlier data in the point-to-bar task, we removed data outside the area defined by ±2 standard deviation of hand position at movement end in the point-to-point task. The screening process did not affect the results for significant magnitude relation of the hand’s viscoelasticity. We calculated deviation of hand position *Δ**P***, velocity *Δ**V***, acceleration *Δ**A***, and force *Δ**F*** from perturbation onset. Using mean values of *Δ**P***and *Δ**F*** across a stable constant phase (the pink area shown in Fig. 4c), we defined a hand stiffness matrix ***K*** as


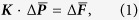


where the overline denotes mean value. The stiffness matrix ***K*** was estimated through a least-squares method. The inertia matrix ***M*** and viscosity matrix ***C*** were also defined as


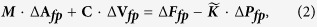


where 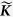 is estimated stiffness matrix. The subscripts *fp* denote deviation data at the dynamic (ramp-up) phase, i.e., at a time when the first peak of hand force appears after perturbation onset (vertical arrow shown in Fig. 4c). By the least-squares method, we estimated ***M*** and ***C*** for each task condition. To stabilize the estimation, we calculated mean inertia matrix 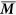 across task conditions. Then we re-estimated hand viscosity matrix ***C*** using the following equation:


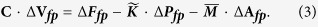


To graphically represent the viscoelastic matrix, we used ellipsoidal representation^25^. This estimation method assumed that the hand viscoelasticity at movement end does not change with time, since the applied perturbation had quite small amplitude and short duration. In addition, the estimations of hand stiffness were difficult in the dynamic phase and for the hand viscosity at steady state by mechanical perturbation. Therefore, we estimated hand stiffness and viscosity at different time points.

## Additional Information

**How to cite this article**: Togo, S. *et al*. Control strategy of hand movement depends on target redundancy. *Sci. Rep.* **7**, 45722; doi: 10.1038/srep45722 (2017).

**Publisher's note:** Springer Nature remains neutral with regard to jurisdictional claims in published maps and institutional affiliations.
